# Probiotics [LGG-BB12 or RC14-GR1] versus placebo as prophylaxis for urinary tract infection in persons with spinal cord injury [ProSCIUTTU]: a randomised controlled trial

**DOI:** 10.1038/s41393-019-0251-y

**Published:** 2019-02-27

**Authors:** Swee-Ling Toh, Bonsan Bonne Lee, Suzanne Ryan, Judy M. Simpson, Kate Clezy, Laetitia Bossa, Scott Alan Rice, Obaydullah Marial, Gerard Hogan Weber, Jasbeer Kaur, Claire Louise Boswell-Ruys, Stephen Goodall, James Walter Middleton, Mark Tuderhope, George Kotsiou

**Affiliations:** 1 Department of Spinal and Rehabilitation Medicine, Prince of Wales Hospital, Sydney, Australia; 2 School of Public Health, University of Sydney, Sydney, Australia; 3 Neuroscience Research Australia [NeuRA] and the University of New South Wales, Sydney, Australia; 4 Department of Infectious Diseases, Prince of Wales Hospital, Sydney, Australia; 5 Centre for Marine Bio-Innovation, University of New South Wales, Sydney, Australia; 6 The Singapore Centre for Life Sciences Engineering and the School of Biological Sciences, Nanyang Technological University, Singapore, Singapore; 7 Royal Rehabilitation Centre Sydney, Sydney, Australia; 8 Royal North Shore Hospital, Sydney, Australia; 9 Centre for Health Economics Research and Evaluation [CHERE], University of Technology Sydney, Sydney, Australia; 10 John Walsh Centre for Rehabilitation Research, Kolling Institute, Northern Sydney Local Health District, St Leonards, NSW 2065 Australia; 11 Sydney Medical School Northern, University of Sydney, Sydney, Australia

**Keywords:** Randomized controlled trials, Nutritional supplements, Spinal cord diseases, Urological manifestations, Bladder

## Abstract

**Study design:**

Randomised double-blind factorial-design placebo-controlled trial.

**Objective:**

Urinary tract infections (UTIs) are common in people with spinal cord injury (SCI). UTIs are increasingly difficult to treat due to emergence of multi-resistant organisms. Probiotics are efficacious in preventing UTIs in post-menopausal women. We aimed to determine whether probiotic therapy with *Lactobacillus reuteri* RC-14+*Lactobacillus* GR-1 (RC14-GR1) and/or *Lactobacillus rhamnosus* GG+*Bifidobacterium* BB-12 (LGG-BB12) are effective in preventing UTI in people with SCI.

**Setting:**

Spinal units in New South Wales, Australia with their rural affiliations.

**Methods:**

We recruited 207 eligible participants with SCI and stable neurogenic bladder management. They were randomised to one of four arms: RC14-GR1+LGG-BB12, RC14-GR1+placebo, LGG-BB12+ placebo or double placebos for 6 months. Randomisation was stratified by bladder management type and inpatient or outpatient status. The primary outcome was time to occurrence of symptomatic UTI.

**Results:**

Analysis was based on intention to treat. Participants randomised to RC14-GR1 had a similar risk of UTI as those not on RC14-GR1 (HR 0.67; 95% CI: 0.39–1.18; *P* = 0.17) after allowing for pre-specified covariates. Participants randomised to LGG-BB12 also had a similar risk of UTI as those not on LGG-BB12 (HR 1.29; 95% CI: 0.74–2.25; *P* = 0.37). Multivariable post hoc survival analysis for RC14-GR1 only vs. the other three groups showed a potential protective effect (HR 0.46; 95% CI: 0.21–0.99; *P* = 0.03), but this result would need to be confirmed before clinical application.

**Conclusion:**

In this RCT, there was no effect of RC14-GR1 or LGG-BB12 in preventing UTI in people with SCI.

## Introduction

### Background

Alterations in urogenital function following a spinal cord injury (SCI) increase the susceptibility of this population to urinary tract infections (UTIs). As a consequence, all UTIs in people with SCI are considered complicated [1]. The incidence of UTI in people with SCI is ~2 episodes per year [2, 3]. Non-antibiotic alternatives like methenamine-hippurate and cranberry tablets, commonly used to prevent UTIs in this population, are ineffective [4].

Another challenge facing clinicians and persons with SCI to manage UTIs is the high rate of colonisation of multi-resistant organisms (MROs) within the population [5, 6]. Simple oral antibiotics are frequently ineffective, which amplifies the health care and economic costs of treating UTIs in this population. Regular antimicrobial prophylaxis is not recommended as it leads to a two-fold increase in MROs [7]. Hence, it is imperative that a non-antibiotic prevention is found, as not to increase the antimicrobial resistance burden [8].

Probiotics are defined as a preparation containing viable, defined micro-organisms in sufficient numbers, which alter the microflora (by implantation or colonisation) in a compartment of the host and thus exert beneficial health effects [9]. A systematic review of trials in women concluded that *Lactobacillus rhamnosus* GR-1 and *Lactobacillus fermentum* RC-14, delivered either intravaginally or orally, were efficacious in preventing recurrent UTIs [10]. In vitro studies have shown that biosurfactants produced by certain Lactobacilli strains inhibit the adhesion of uropathogenic bacteria to silicone rubber [11]. Clearance of vancomycin-related enterococci in stool after treatment with *L. rhamnosus* GG has also been reported [12]. Thus, literature suggests that probiotics may have a role in preventing UTIs.

### Objectives

There are currently no known studies of oral probiotics and their efficacy in the prevention of UTIs in people with SCI and neurogenic bladder. We propose a randomised controlled trial (RCT) to investigate the effectiveness of combination oral probiotic therapy [*L. reuteri* RC-14+*L. rhamnosus* GR-1 (RC14-GR1) and/or *Lactobacillus* GG+*Bifidobacterium* BB-12 (LGG-BB12) capsules] to prevent UTI in people with SCI compared to placebo. The rationale being that the combination probiotics will have a two-pronged approach of clearing MROs which is highly prevalent in SCI persons as well as in preventing UTIs. MRO clearance is beyond the scope of this manuscript report.

## Methods

ProSCIUTTU was a prospective multi-site randomised, double-blind, double-dummy, placebo-controlled factorial design trial conducted in the state of New South Wales (NSW), Australia [13]. Prior to commencement, approval was granted from a lead human ethics committee covering the eastern seaboard of Australia. Research governance approval was also granted in all hospitals involved in the trial. The trial was registered with the Australian New Zealand Clinical Trials Registry (ACTRN12610000512022). The trial protocol and full methodology has previously been published, therefore we will present a briefer version in this paper [13].

### Participants

Participants were actively recruited from the database of all past and current patients in each of the three specialist SCI units in NSW (Prince of Wales Hospital, Royal North Shore Hospital and Royal Rehabilitation Centre Sydney), including their rural affiliations. Participants were over 18 years of age, with SCI and stable neurogenic bladder management. Participants agreed not to take any other probiotics in addition to the allocated intervention during the course of the study. Exclusion criteria were complex bladder disturbances requiring surgical intervention, known urinary tract calculi, having received bladder education within the last 4 weeks, pre-existing infection on intervention commencement, known long-standing osteomyelitis, long-term antibiotic therapy, adverse reaction to yoghurt products, severe renal or hepatic failure, full mechanical ventilation and immunosuppression. Participants provided written informed consent before enrolment.

### Interventions

Participants were enroled for a 6-month study period, which included 24 weeks of treatment. Each randomised participant was required to take two capsules orally each day consisting of either:

1. Group 1: RC14-GR1 (concentration per capsule is 5.4 × 10^9^ colony-forming units)+LGG-BB12 (concentration per capsule is 7 × 10^9^ colony-forming units)
2. Group 2: RC14-GR1 (concentration as above)+matched placebo (no LGG-BB12)
3. Group 3: LGG-BB12 (concentration as above)+matched placebo (no RC14-GR1)
4. Group 4: Double matched placebo capsules (no RC14-GR1 and no LGG-BB12)
5. To ensure that there was no degradation of probiotics during the entire duration of the trial, the capsules were stored at −25 °C and tested at 12 monthly intervals for organism numbers throughout the study.

### Outcomes

**Fig. 1:**
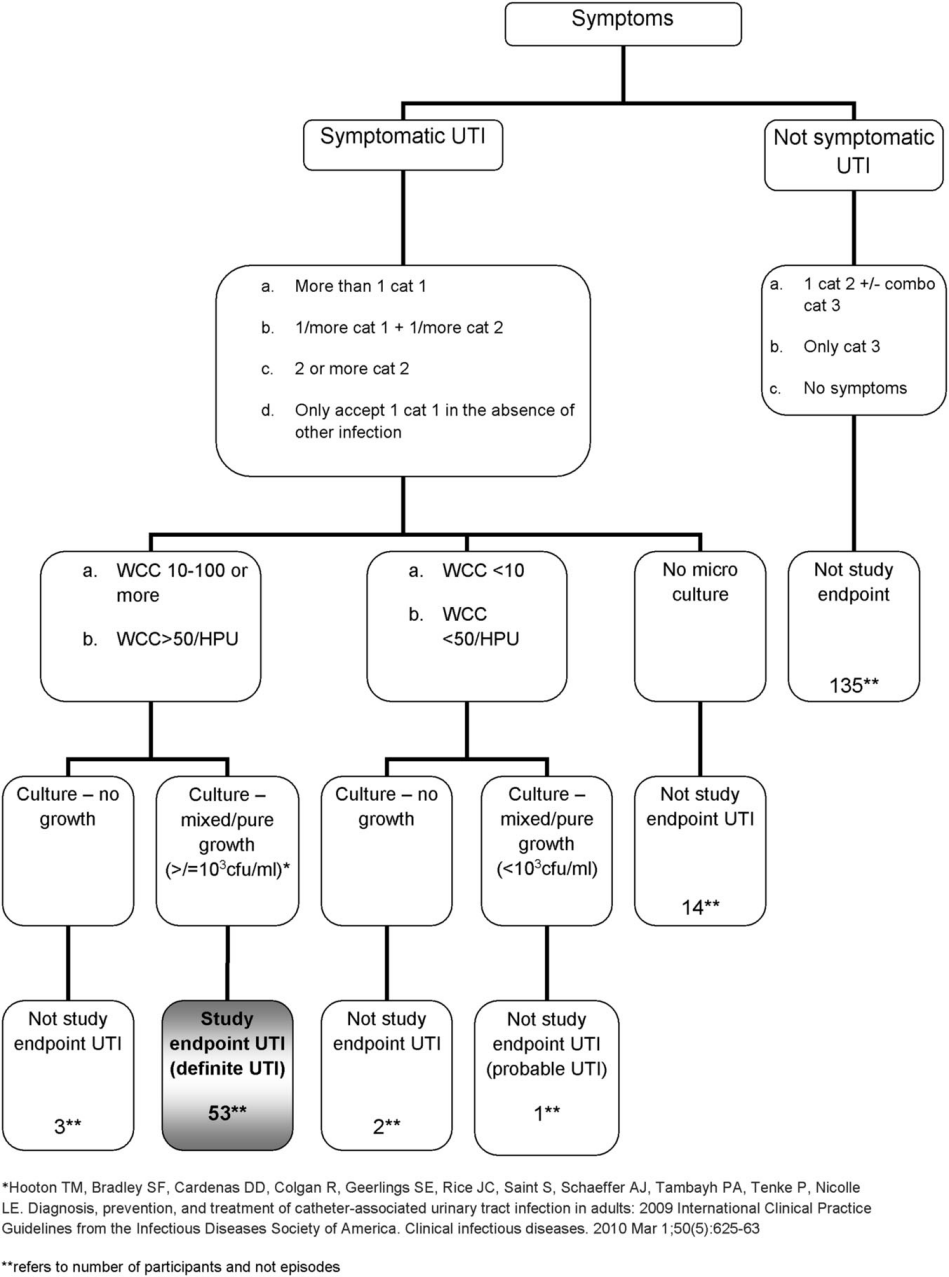
Definition of primary endpoint UTI for ProSCIUTTU

Primary outcome measure is the time from randomisation to occurrence of first *symptomatic* UTI (primary endpoint) (Fig. 1). Symptomatic UTI is not well -defined in the SCI literature [14]. UTI for the purpose of our study was only based on a combination of symptom(s) and microbiological analysis of urine. Our criteria was based on a consensus statement by the National Institute on Disability and Rehabilitation Research and a previous trial [4, 15]. Supplementary Table 1 outlines the category of symptoms for the algorithm. Participants who did not experience a symptomatic UTI were censored at 6 months or when they withdrew from the trial.

Participants were assessed at baseline, 3 and 6 months, supported by telephone calls every 2 weeks to confirm intercurrent symptomatic UTI status. To evaluate quality of life, the Short Form Health Survey (SF-36) was conducted at baseline, study endpoint and 6 months. Microbiological swabs of the rectum, nose and groin and a urine culture were obtained at baseline, 3 months and 6 months. These samples were analysed at a central laboratory and are beyond the scope of this manuscript report. A urine culture was also performed if participants developed symptoms of UTI. We requested at all occasions for urine to be collected from a new single use catheter or new suprapubic catheter. The endpoint urine cultures were analysed at the participant’s local microbiological laboratory due to logistics and clinical reasons.

All participants were also given information about symptoms of a UTI, contact numbers of researchers and the participant’s SCI unit so they can contact their SCI physician.

### Sample size

In our previous RCT with the same study population [4], 45% of participants had a symptomatic UTI within 6 months. To have 80% power to detect [at 5% two-sided significance level] a reduction to 30% in the treatment group requires a total sample size of 350. Allowing for a 5% loss to follow-up a final sample size of 372 is required, with 93 participants randomly allocated to each of the four study groups.

### Randomisation

A simple computer-generated randomisation protocol was used. Randomisation was stratified by bladder management type (indwelling urethral/suprapubic vs. intermittent catheters vs. condom drainage/reflex voiding) and inpatient/outpatient status. Randomisation occurred after informed consent and baseline measures were obtained from the participant. The washout period and compliance check with placebo, as stated in the protocol, were carried out for the first 14 patients and all were found to be 100% compliant. However, due to logistical difficulties and to reduce travelling costs for researchers, the protocol was amended and the washout and compliance check were abandoned. One central pharmacy was responsible for the assignment and distribution of the intervention for the entire study.

### Blinding

All four treatment regimens were indistinguishable by appearance and taste. All clinical staff, researchers and participants remained blind to treatment allocation throughout the trial. Clinicians (BBL, GHW, JK, KC and S-LT) who had patients in the study were kept blinded at all times including during the blinded analysis phase. Microbiological assessments were available to investigators and were released if clinicians on the study felt it would assist with medical management. All staff was blinded to allocation for the assessment of symptomatic outcomes. An audit of randomisation, product allocation and dispensing stock was performed at the completion of the study.

### Statistical methods

Analysis of all outcomes was by intention to treat. Primary outcome was analysed using survival analysis. The log-rank test statistic was used to assess the significance of the unadjusted effect of a variable in preventing UTI. Kaplan–Meier survival curves, hazard ratios (HR) and their 95% confidence intervals (CI) were used to summarise results of the effect of RC14-GR1 and LGG-BB12 on time to primary endpoint. Cox regression modelling was performed to test the effect of each treatment while allowing for the other. To determine which variables were clinically correlated with UTI in the SCI population, a survey was sent out to four investigators (BBL, GHW, KC and SAR). They were requested to rate the association of a list of variables collected for the trial as strong, moderate or weakly associated with UTI. The variables gender, inpatient status, completeness of injury (American Spinal Injury Association Impairment Scale Grade A) [16], bladder management, time since injury, current urinary tract stone and UTI in the last 6 months were considered to be moderate to strongly associated with UTI. Analysis was performed using SAS 9.3.

## Results

### Baseline data

Study participants were recruited from April 2011 to February 2014.

**Fig. 2:**
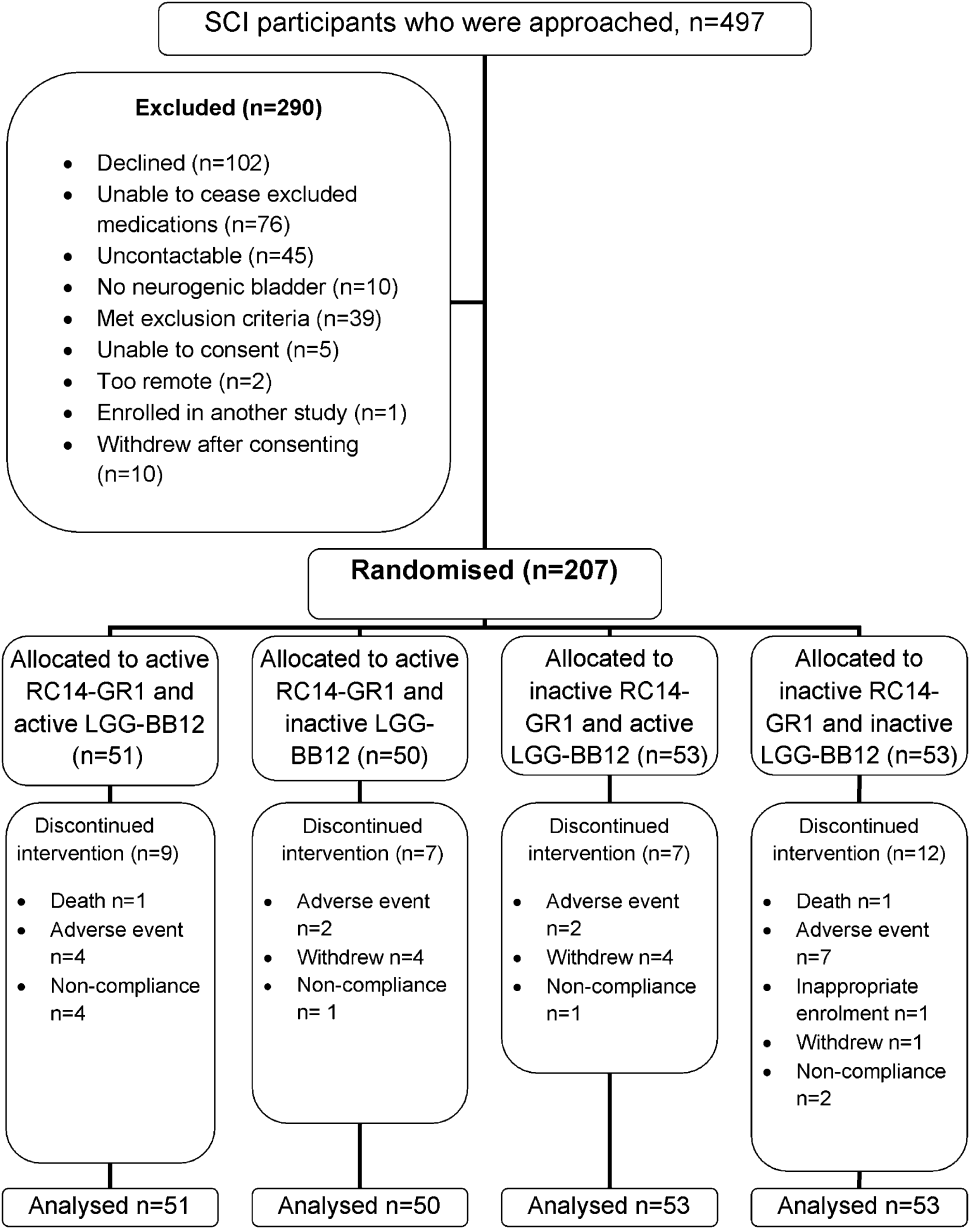
Consort participant flow diagram

Four-hundred and ninety-seven participants were approached via correspondence, telephone contact or in person. Three-hundred and fifty participants were interested and were screened for eligibility. Of the 350 participants screened, 10 did not have neurogenic bladder and 76 either did not want or could not cease excluded medications like antibiotics and their current probiotics (Fig. 2—participant flow). Thirty-nine were excluded due to criteria such as complex bladder disturbance or chronic pressure ulcers.

All 207 participants who were randomised were analysed by original group assignment (intention to treat). Participants had a mean age of 49.1 years (range 19–82 years) and were predominantly male (79%). Forty-seven percent were tetraplegics and 51% had complete injury (American Spinal Injury Association Impairment Scale Grade A). Sixty-percent of participants had a suprapubic or indwelling urethral catheter as part of neurogenic bladder management. The median time since SCI was 7 years (range 10 days to 61 years).

There were no clinically important differences between the treatment groups at baseline in the characteristics measured (Table 1).

**Table 1:** Participant demographic characteristics by treatment group

		RC14-GR1		LGG-BB12	
		RC14-GR1	No RC14-GR1	LGG-BB12	No LGG-BB12
		*n* (%)	*n* (%)	*n* (%)	*n* (%)
Characteristics	*N*	101	106	104	103
*Participant location at recruitment*					
Inpatient		19(19)	21(20)	22(21)	18(17)
Outpatient		82 (81)	85(80)	82(79)	85(83)
*Gender*					
Female		18(18)	26(25)	18(17)	26(25)
Male		83(82)	80(75)	86(83)	77(75)
*Bladder management*					
Indwelling catheter		9(9)	12(12)	12(12)	9(8)
Suprapubic catheter		51(50)	51(48)	49(47)	53(51)
Clean intermittent catheters		34(34)	37(35)	38(36)	33(32)
Reflex voiding (±condom)		7(7)	6(5)	5(5)	8(8)
*Level of injury*					
Cervical		46(45)	51(48)	48(46)	49(44)
Thoracic		43(43)	50(47)	48(46)	45(47)
Lumbar		12(12)	5(5)	8(8)	9(9)
*Completeness of injury* ^a^					
Complete		53(52)	53(50)	55(53)	51(50)
Incomplete		48(48)	53(50)	49(47)	52(50)
*Presence of urinary stone*					
None		98(97)	98(92)	97(93)	99(96)
Present		3(3)	8(8)	7(7)	4(4)
*Age (years)*					
<25		6 (6)	9(8)	8(8)	7(7)
25 to <45		33(33)	34(32)	37(36)	30(29)
45 to <65		48(47)	43(41)	44(42)	47(46)
>65		14(14)	20(19)	15(14)	19(18)
*Time since injury*					
<2 years		27(27)	30(28)	29(28)	28(27)
2–20 years		52(51)	43(41)	48(46)	47(46)
>20 years		22 (22)	33(31)	27(26)	28(27)
*AIS classification* ^a^					
A		53(52)	53(50)	55(53)	51(50)
B		17(17)	19(18)	22(21)	14(14)
C		14(14)	19(18)	14(13)	19(18)
D		17(17)	15(14)	13(13)	19(18)
E		0	0	0	0
*Urinary tract infection in the last 6 months prior to trial*					
0		38(37)	44(41)	49(47)	33(32)
1		31(31)	23(22)	19(18)	35(34)
2 to 5		26(26)	33(31)	32(31)	27(26)
More than 5		6(6)	6(6)	4(4)	8(8)
*Hospitalisation prior to trial in last 6 months*					
0		91(90)	91(86)	91(87)	91(88)
1		8(8)	12(11)	9(9)	11(11)
2–5		1(1)	3(3)	3(3)	1(1)
More than 5		1(1)	0	1(1)	0
*General practitioner visit for urinary tract infection before trial*					
None		60(59)	65(61)	69(66)	56(54)
1		22(22)	12(11)	10(10)	24(23)
2–5		13(13)	26(25)	19(18)	20(20)
5 or more		6(6)	3(3)	6(6)	3(3)

^a^American Spinal Injuries Association (ASIA) Impairment Scale (AIS) for neurological classification of SCI definition [16]

### Outcomes and estimation

Fifty-three of 207 participants met study endpoint UTI, as follows: 14 in Group 1, 8 in Group 2, 15 in Group 3 and 16 in Group 4. A further 20 participants had symptomatic but not study endpoint UTI; 5 of these had fever. Of the 53 study endpoint UTI participants, 22 had febrile UTIs. As a consequence of their endpoint UTI, 3 participants developed urosepsis, one participant was diagnosed with pyelonephritis and one participant was diagnosed with orchitis. All except one participant who met the study endpoint utilised catheters to empty their bladder. In terms of UTI recurrence in the 53 participants, 11% (6/53) had a subsequent study endpoint UTI and 6% (3/53) had a further two study endpoint UTIs within the 6-month duration of the study.

For urine cultures that met study endpoint, 36/53 urine grew gram negative organisms with the predominant species being *Escherichia coli*.

**Fig. 3:**
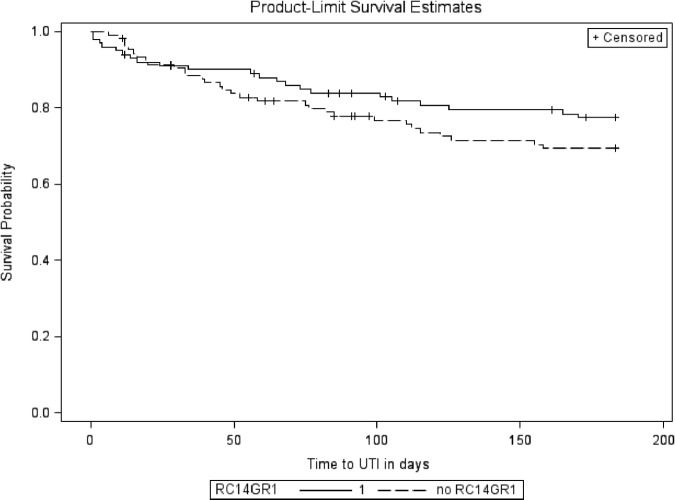
Kaplan–Meier curve for RC14-GR1 compared to no RC14-GR1

**Fig. 4:**
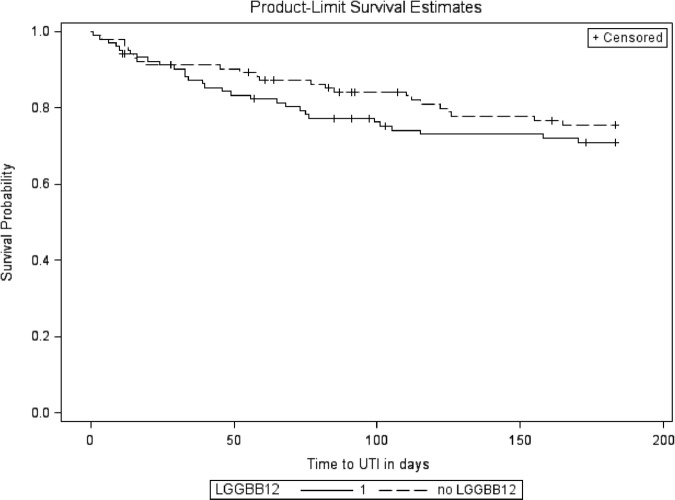
Kaplan–Meier curve for LGG-BB12 compared to no LGG-BB12

After 6 months of oral probiotics therapy, the unadjusted analysis (Table 2) showed no significant effect on symptomatic UTI of RC14-GR1 (HR 0.71, 95% CI: 0.41–1.23; *P* = 0.22) or LGG-BB12 (HR 1.27, 95% CI: 0.74–2.18; *P* = 0.39). The Kaplan–Meier survival curves for participants on active treatment with RC14-GR1 (groups 1 and 2 combined) and those not on RC14-GR1 (groups 3 and 4) are shown in Fig. 3. The curves for those on active treatment with LGG-BB12 (groups 1 and 3) and those not on LGG-BB12 (groups 2 and 4) are shown in Fig. 4.

**Table 2:** Unadjusted effects of all pre-specified covariates, including RC14-GR1 and LGG-BB12, on UTI-free survival

Covariates	Hazard ratio (95% CI)	*P*-value (log rank)
RC14-GR1		
Placebo	1	
Active	0.71 (0.41, 1.23)	0.22
LGG-BB12		
Placebo	1	
Active	1.27 (0.74, 2.18)	0.39
Participant location at recruitment		
Inpatient	1	0.40
Outpatient	0.76 (0.40, 1.45)	
Gender		
Female	1	0.82
Male	1.08 (0.56, 2.10)	
Bladder management		
Indwelling catheter	1	0.40
Suprapubic catheter	0.68 (0.29, 1.57)	
Intermittent catheter	0.83 (0.35, 1.97)	
Reflex voiding (±condom)	0.21 (0.03, 1.70)	
Level of injury		
Cervical	1	0.63
Thoracic	0.84 (0.48, 1.47)	
Lumbar	0.59 (0.18, 1.95)	
Completeness of injury^a^		
Complete	1	0.052
Incomplete	0.58 (0.33, 1.01)	
Current renal/bladder stone		
None	1	0.99
Present	0.99 (0.31, 3.17)	
Age (years)	0.99 (0.97, 1.01)	0.43
Time since injury (years)	0.99 (0.98, 1.02)	0.64
ASIA level^a^		
A	1	0.17
B	0.67 (0.31, 1.44)	
C	0.73 (0.34, 1.58)	
D	0.35 (0.12, 0.98)	
UTI 6 months prior to trial		
No	1	0.002
Yes	1.21 (1.08, 1.35)	

^a^American Spinal Injuries Association (ASIA) Impairment Scale (AIS) for neurological classification of SCI definition [16]

The only statistically significant predictor of a future UTI was whether there was a UTI in the preceding 6 months (log-rank *χ*^2^ = 12.24, df = 2; *P* = 0.002), and there was some evidence that an incomplete injury may be protective against UTI (*P* = 0.052). There was no statistically significant relationship between UTI-free survival and inpatient/outpatient status, bladder management, gender, age and time since injury. As the number of participants who were found to have urinary stones was low (eleven), it was difficult to predict the effect of this variable on UTI-free survival.

Multivariable analysis using Cox proportional hazards regression (Table 3), adjusting for pre-specified variables gender, inpatient status, bladder management, completeness of injury, time since injury and UTI in six months prior to study, showed no statistically significant effect of RC14-GR1 compared to no RC14-GR1 (HR 0.67, 95% CI: 0.39–1.18; *P* = 0.17) or of LGG-BB12 compared to no LGG-BB12 (HR 1.29, 95% CI: 0.74–2.25; *P* = 0.37).

**Table 3:** Adjusted effects of RC14-GR1 and LGG-BB12 treatments and other covariates on UTI-free survival

Covariates	Hazard ratio (95% CI)	*P*-value (Likelihood ratio test)
RC14-GR1		
Placebo	1	0.17
Active	0.67 (0.39, 1.18)	
LGG-BB12		
Placebo	1	0.37
Active	1.29 (0.74, 2.25)	
Participant location at recruitment		
Inpatient	1	0.57
Outpatient	0.79 (0.34, 1.81)	
Gender		
Female	1	0.78
Male	1.10 (0.55, 2.20)	
Bladder management		
Indwelling catheter	1	0.95
Suprapubic catheter	0.67 (0.26, 1.76)	
Intermittent catheter	0.78 (0.31, 1.98)	
Reflex voiding (±condom)	0.34 (0.04, 3.01)	
Completeness of injury^a^		
Complete	1	0.03
Incomplete	0.52 (0.29, 0.94)	
Time since injury	0.99 (0.97, 1.02)	0.48
UTI 6 months prior to trial	1.24 (1.10, 1.40)	0.002

^a^American Spinal Injuries Association (ASIA) Impairment Scale (AIS) for neurological classification of SCI definition [16]

### Ancillary analysis

**Fig. 5:**
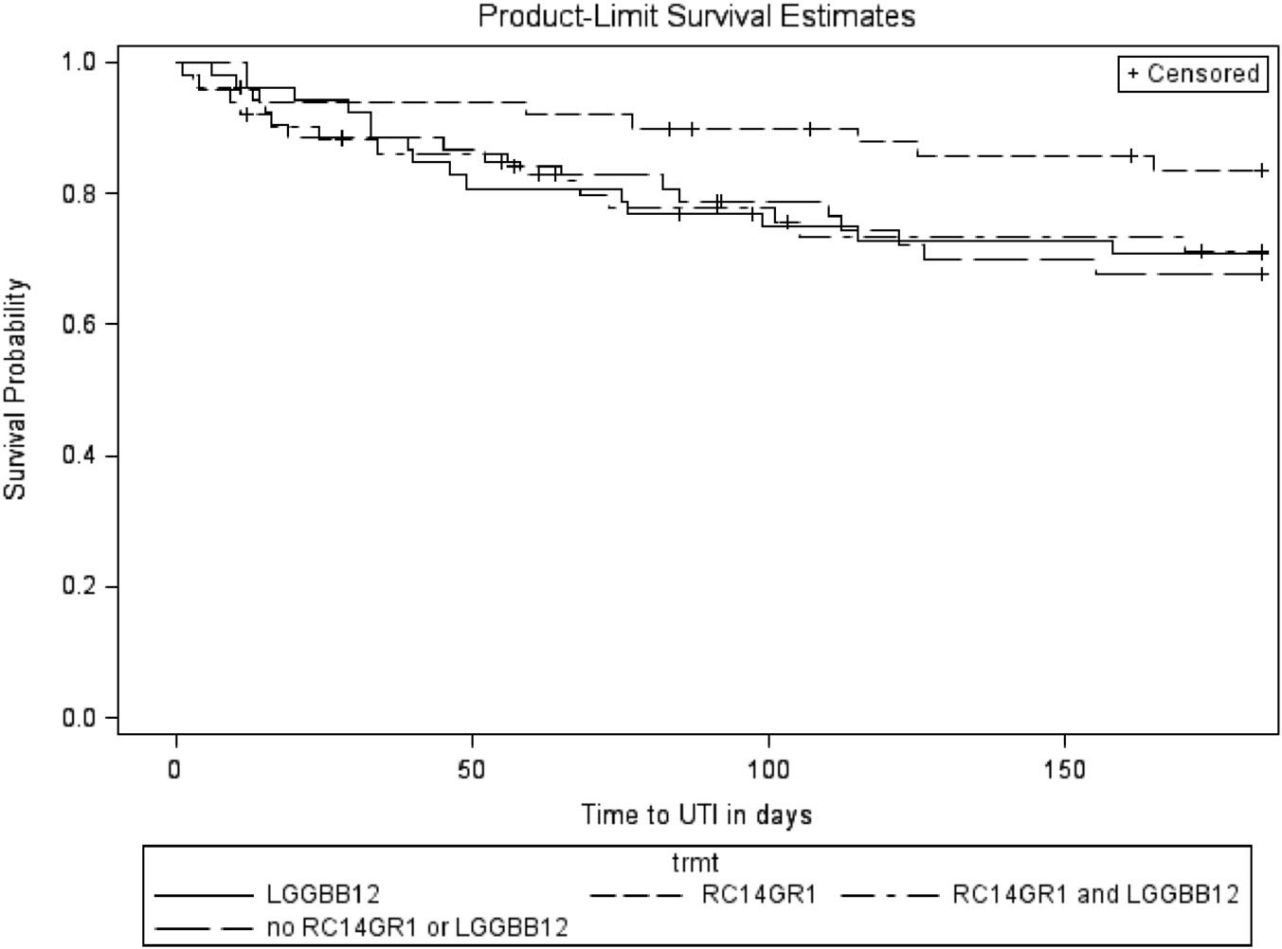
Kaplan–Meier curve for RC14-GR1 vs. three other treatment groups

The Kaplan–Meier survival curves appeared to show longer UTI-free survival for RC14-GR1 by itself (Group 2) than the other groups (Fig. 5). Post hoc multivariable analysis was therefore carried out for RC14-GR1 alone pooled against the three other treatment groups using Cox proportional hazards regression. There was a statistically significant effect of RC14-GR1 *alone* after adjusting for the same covariates (HR 0.46, 95% CI: 0.21–0.99; *P* = 0.03).

Analysis was conducted for the 184 participants who answered the SF-36 questionnaire at baseline and end of treatment. There was no statistically significant difference between the baseline and end-of-treatment quality of life of participants in any of the trial groups under the eight different domains of the SF-36 (physical functioning, role physical, role emotion, social functioning, pain, mental health, vitality and general health).

### Harms

Side effects from either intervention were infrequent. The double placebo group appeared to have more adverse events than the other groups. The majority of adverse events were due to bowel complaints, such as bowel accidents and increased frequency of bowel movement. Other adverse events reported were symptoms of UTI, abdominal cramps, blocked urinary catheter and groin rash. Two deaths in the study were unrelated to trial intervention as they were due to respiratory failure and biliary sepsis (Supplementary Table 2).

## Discussion

In this double-blind, double-placebo RCT, there was no effect of RC14-GR1 or LGG-BB12 in preventing UTI in people with SCI using a factorial design. The post hoc analysis of RC14-GR1 (alone) suggests the potential that RC14-GR1 may be a beneficial organism for preventing UTI when used *alone*. A priori, this study was not designed to test this hypothesis.

Our results for LGG-BB12 are consistent with anything from a protective effect against UTI (lower 95% confidence limit for HR: 0.74) to increasing the risk of UTI (upper 95% confidence limit for HR: 2.18). The possibility of LGG-BB12 increasing the likelihood of a UTI would indicate that not all probiotics are necessarily beneficial in all circumstances, which raises questions about mixed organism probiotics being used in the community to prevent UTI. RC14-GR1 is an organism with prior urinary prophylactic data [10], while LGG is an organism where there is more data about MRO colonisation reduction [12]. More task selectivity for probiotic organisms is likely to be necessary, as well as a better understanding of the dosages required for UTI prevention.

Our results confirm the relationship between previous UTIs and increased likelihood of recurrences. In our cohort, if participants had at least one UTI in the preceding 6 months, their risk of having a subsequent UTI was increased by 21% in the following 6-month period (Tables 2 and 3). This concurs with findings from the previous SINBA study [4]. One critique of our study could be the high number of participants (>60%) utilising either an indwelling urethral or suprapubic catheter. Indwelling urethral and suprapubic catheters have been associated with higher rates of urinary tract complications including UTIs [17]. Intermittent self-catheterisation rates in this cohort were only 30%. However, Cameron reported that only 20% of SCI patients on intermittent catheterisation remained on this form of management after 30 years with the majority reverting to use of an indwelling urethral/suprapubic catheter [18].

One of the strengths of the study is obtaining urinary microbiological confirmation for participants who met the endpoint criteria for “symptomatic UTI”. This is despite their endpoint urine analysis not being performed in the central laboratory used by the study. Due to logistical and clinical reasons, the endpoint urine culture was analysed at the participant’s local microbiological laboratory. There was one participant who did not meet urinary microbiological criteria for “symptomatic UTI”. That participant was classified as meeting study endpoint based on symptoms and presence of pyuria in the urine analysis. This decision was made following a discrepancy between two blinded assessors (S-LT and BBL) leading to verification by a third blinded assessor (KC). There was another participant who met endpoint criteria but was considered not appropriate by KC as the participant had hypothermia instead of a fever, no Category 2 urinary symptoms and a fractured neck of femur to account for autonomic dysreflexia and abdominal pain.

The overall event rate was also much lower than expected from our previous research [4]. This could be due to the stringency of our endpoint criteria, needing to meet both symptoms and microbiological confirmation. In our previous trial, endpoint was based on symptoms and positive urine dipstick or presence of pyuria. For this trial we wanted to achieve best practice in UTI treatment. If we did not insist on urinary microbiological confirmation, we would have had an additional 20 participants meeting endpoint criteria.

In our sample size calculation, we predicted 45% of participants in the control group would develop a symptomatic UTI within 6 months but only 26% of the total participants did. With 103 per group (active RC14-GR1 vs. no RC14-GR1 OR active LGG-BB12 vs. no LGG-BB12) we had 80% power to detect a reduction from 34% UTI in the control to 17% in the treated group at the two-sided 5% significance level (these rates would give 53 expected UTIs in total). By this calculation the treatment would have needed to halve the UTI rate, not just reduce it by 33% proportionately (from 45% to 30%).

The major limitation of our study was the failure to recruit the targeted 372 participants. The main reason was insufficient funding for such a geographically dispersed cohort of participants. Trial funding only accounted for participants who lived in close proximity (around 35 km) to each of the SCI units located in metropolitan Sydney. We ended up recruiting participants in several regional and remote areas outside of metropolitan Sydney. This was partly due to competition from other concurrent SCI trials which limited access to eligible metropolitan participants. In retrospect, more detailed costing should have been performed in order to apply for more appropriate funding. Travel costs consumed a significant amount of resources. The cost of microbiological analysis also exceeded the budget. Some potential study participants were reluctant to give up yoghurt or cease their current probiotics or antibiotic regime for the duration of study. It is possible that more recruitment success could have been achieved by involving additional national or international partners. Nevertheless, this is the largest randomised trial of oral probiotics in a neurogenic bladder population.

Another limitation of our study is not following up participants after trial completion (e.g. 3 or 6 months) to see whether their incidence of UTI changed.

We cannot compare the results of our study to other studies as there is no other RCT of oral probiotics in the neurogenic bladder population [19]. We are aware of a Cochrane review reporting low evidence of probiotics in preventing UTIs in adults and children [20]. Kontiokari has previously reported in an RCT that cranberry was more effective than a *Lactobacillus* GG drink in preventing UTIs in women [21]. Beerepoot reported that oral capsules of *L. rhamnosus* GR-1 and *L. reuteri* RC-14 taken for 12 months did reduce the mean number of symptomatic UTI in post-menopausal women, however did not meet the non-inferiority criteria when compared to trimethoprim-sulfamethaxole [22].

We have not been able to demonstrate that RC14-GR1 or LGG-BB12 is effective in preventing UTI in people with SCI. There is the possibility that RC14-GR1 when used alone may be beneficial, but this finding is hypothesis generating only, due to post hoc analysis. Our study results indicate that the clinical use of probiotics should be more selective and specific. Further research into dosages as well as task selectivity of probiotics should be conducted in the future.

### Data archiving

The UTI symptomatic data is being made available to the ISCoS Datasets committee in a de-identified manner to assist with ISCoS UTI data definitions and Bowel data definitions. Records will be archived at NEURA for 15 years.

#### Registration

Australian New Zealand Clinical Trials Registry [ACTRN12610000512022]. http://www.anzctr.org.au.

#### Protocol

Open access from BMC Urology https://bmcurol.biomedcentral.com/articles/10.1186/s12894-016-0136-8.

## Supplementary information

Table 1- Category of symptoms for defintion of UTI as primary endpoint for ProSCIUTTU

Table 2- Number of participants experiencing adverse events
