# Supplementary material for: Probiotics [LGG-BB12 or RC14-GR1] versus placebo as prophylaxis for urinary tract infection in persons with spinal cord injury [ProSCIUTTU]: a randomised controlled trial
Source: Spinal Cord. 2019 Feb 27;57(7):550–61. doi: 10.1038/s41393-019-0251-y (PMC6760555; doi:10.1038/s41393-019-0251-y)
Supplement: Supplementary file 1 — Table 1- Category of symptoms for defintion of UTI as primary endpoint for ProSCIUTTU [file 41393_2019_251_MOESM1_ESM.pdf]

Supplementary Table 1: Category of symptoms for definition of Symptomatic UTI as primary endpoint for ProSCIUTTU (refer to Figure 1)

Use the following table to assess “Category 1”, or Two “Category 2” and any “Category 3” Symptoms: All symptoms should be asked in each category

"Category 1" Symptoms: *	**“Category 2” Symptoms**: *<br>Two or more	**“Category 3” Symptoms:**<br>In themselves not enough to lead to treatment but recorded for International Datasets Compatibility
• **Temperature**:<br>Greater than 38° C core<br><br>Greater than 37.5° C per axilla<br><br>• New or increasing symptoms of **Autonomic Dysreflexia**, as detected by any of the following signs: *Pulse < 50 or increased flushing or sweating or headache AND increased B.P Diastolic or Systolic > 25% usual baseline.*	• Increased Frequency of Muscle Spasms or spasticity<br>• Failure of usual control of urinary incontinence-<br>*any of the following constitutes fulfillment of this category*<br>- Bladder Spasm<br>- Urinary frequency or need for increased catheterization<br>- Urinary Retention<br>- Urinary Urgency<br>- Leaking around catheter site or per urethra if have suprapubic catheter<br>• New Scrotal/Loin/Abdominal Discomfort unexplained by other pathology - *any of the following constitutes fulfillment of this category*<br>- Abdominal Pain<br>- Bladder/Suprapubic Pain<br>- Loin/Back Pain<br>- Scrotal Pain<br>- Dysuria	*any of the following constitutes fulfillment of this category*<br>- Anxiety/uneasiness<br>- Feeling tired<br>- Feeling sick<br>- Arthralgias/Body Aches<br>- Chills<br>- Diaphoresis/sweating<br>- Cloudy Urine<br>- Foul smelling urine<br>- Blood in urine *haematuria*<br>- Catheter blockage

*Content adapted and modified from Box 1 of Spinal-Injured neuropathic bladder antisepsis (SINBA) trial (4)
